# A meta-analysis for efficacy and safety evaluation of transcatheter left atrial appendage occlusion in patients with nonvalvular atrial fibrillation

**DOI:** 10.1097/MD.0000000000004382

**Published:** 2016-08-07

**Authors:** Zhonghai Wei, Xinlin Zhang, Han Wu, Jun Xie, Qing Dai, Lian Wang, Biao Xu

**Affiliations:** Department of Cardiology, Drum Tower Hospital, Medical School of Nanjing University, Nanjing, Jiangsu, China.

**Keywords:** catheter, left atrial appendage, meta-analysis, occlusion, thrombus

## Abstract

**Objectives:**

This meta-analysis was conducted to evaluate the efficacy and safety of transcatheter left atrial appendage (LAA) occlusion in patients with nonvalvular atrial fibrillation.

**Methods:**

The randomized controlled trials (RCT) or observational studies with any transcatheter LAA occlusion devices were searched in PubMed, Embase, and Cochrane library from inception to November 2015. The incidence rates from individual studies were combined to evaluate the procedural efficacy and safety, including all-cause death, cardiac/neurological death, stroke, transient ischemic attack (TIA), thrombosis, hemorrhagic complications, and pericardial effusion/tamponade.

**Results:**

Thirty-eight studies involving 3585 patients and 6 different occlusion devices were eligible for our inclusion criteria. The procedural failure rate for LAA closure was 0.02 (95% CI: 0.02–0.03). The all-cause mortality was 0.03 (95% CI: 0.02–0.03) and cardiac/neurological mortality was 0 (95% CI: 0.00–0.01). The stroke/TIA rate was estimated only 0.01 (95% CI: 0.01–0.01). The incidence of thrombus on devices was 0.01 (95% CI: 0.01–0.02). The major hemorrhagic complication rate was estimated 0.01 (95% CI: 0.00–0.01). Pericardial effusion/tamponade was estimated 0.02 (95% CI: 0.02–0.03). No heterogeneity was observed for above pooled estimates (*I*^2^ = 0). In devices subgroups analysis, the all-cause mortality and cardiac/neurological mortality of PLAATO group were the highest (*P* = 0.01 and *P* < 0.01 respectively), whereas the incidence of thrombus on devices in the ACP group was the highest (*P* < 0.01). In follow-up period subgroups analysis, there were significant differences in all-cause death, stroke/TIA, major hemorrhage, and pericardial effusion/tamponade events between the shorter and longer follow-up period subgroups (*P* < 0.05). However, the differences among the subgroups were numerically small.

**Conclusions:**

the pooled data demonstrated that transcatheter LAA occlusion was effective and safe in the patients with nonvalvular atrial fibrillation who were not suitable for lifelong antithrombotic therapy.

## Abstract 2

Supplemental Digital Content is available in the text

## 1 Introduction

Atrial fibrillation (AF) is a common arrhythmia, which affects ∼1  to  2% of the general population. Structural and electrophysiological remodeling is essential for AF, which is due to complicated factors. In fact, metabolic triggers, such as insulin and glucose homeostasis, may alter inflammation and oxidative stress, subsequently affecting channels activity and inducing a proarrhythmic state.^[1–5]^ Stroke, as a main cause of cardiovascular death, is prevalent remarkably with aging and ∼20% stroke is attributed to AF. The people with AF are likely to have 5-fold risk of stroke than the people without AF.^[6]^ Notably, subclinical episode of AF, which is related to autonomic dysfunction, occurs more frequently in type 2 diabetic patients and also increases the risk of silent cerebral infarct and stroke.^[7,8]^ Stroke caused by AF usually results in long-term death, disability, and poor quality of life. Antithrombotic therapy is therefore considered as the first issue in AF patients. Vitamin K antagonists (VKA) have been used for prevention of stroke in AF for several decades. The antithrombotic effects of VKA have been confirmed in comparison with placebo and antiplatelet drugs.^[9,10]^ Novel oral anticoagulants (NOAC), such as dabigtran, rivaroxaban, were identified noninferior or superior to warfarin in prevention of stroke, but without increasing risk in hemorrhagic complications.^[11,12]^ However, the therapy of anticoagulant would make the patients under long-term hemorrhagic risk, especially for the aged patients >80 years. It was reported that the incidence of severe hemorrhage was 13.1/100 person-years.^[13]^ In the real world, a considerable proportion of AF patients are not suitable for or not willing to long-term anticoagulant. Thus, transcatheter left atrial appendage (LAA) occlusion is a probable alternative in stroke prevention for this subset of the nonvalvular AF patients. The LAA occlusion device was designed to occluded the orifice of LAA, where is the origin of >90% of thrombi. There have been so far 2 randomized controlled trials (RCT), PROTECT AF, and PREVAIL, which have identified the noninferiority of LAA closure to long-term use of warfarin in stroke prevention.^[14,15]^ Nonetheless, the procedure of LAA occlusion is risky and challenging and the safety is always the concern of interventionists. In PROTECT AF and PREVAIL study, the noninferiority of primary safety endpoints were not both achieved.^[14,15]^ Numerous observational studies have focused on the subjects, many of which involved relative small sample size, however. We performed the meta-analysis, taking advantage of the information from all the relevant studies to evaluate the efficacy and safety outcomes of LAA occlusion.

## 2 Methods

### 2.1 Study search

We searched the relevant studies without language limitation in PubMed, Embase, and Cochrane library (∼ November 2015) using the following Keywords: “left atrial appendage,” closure, occlu^∗^, limited with human species. Additional studies were sought by reviewing the reference lists of eligible studies. Duplicated articles from different databases were excluded after initial search. Conference abstracts or articles could be included if the index data were accessible. We have also attempted to contact the authors in order to acquire the full-text of the studies if the data were not accessible online. The ethical approval was not necessary because the present study just analyzed the data extracted from the previous studies and did not include any patients.

### 2.2 Study selection

The eligible studies were identified by 2 investigators independently (ZW and XZ) and the disagreement was solved by discussion. The criteria for inclusion were well-designed RCT; observational studies, including cohort studies, case-control studies; (3) transcatheter LAA closure with different devices; mortality and stroke after procedure were assessed. Criteria of exclusion were case report; study population <5 patients; LAA closure with surgical procedure; mortality or stroke was not reported; study not written in English.

### 2.3 Quality assessment

The methodological quality of the eligible studies was assessed in the following aspects: randomization during the allocation, blinding during the procedure, concealment during the assignment and loss of follow up. The quality of included observational studies was evaluated with the Newcastle–Ottawa scale (NOS) criteria.

### 2.4 Data extraction

The data extraction was performed by 2 independent investigators and disparity was solved by discussion. The data included design of the trials, treatment regimens, devices type and size, events after procedure, including all-cause death, cardiac/neurological death, stroke, thrombus on devices, hemorrhage complications and pericardial effusion/tamponade.

### 2.5 Statistical analysis

Summary results were presented as incidence rate of the events (ratio of events number to patient number) and 95% confidence interval (CI). We combined the individual studies using fixed-effects models based on inverse variance method. Due to the existence of extreme values, pooled estimate was calculated after Freeman–Tukey double arcsine transformation for individual studies to stabilize the variance. *I*^2^ was calculated to assess the heterogeneity and *I*^2^ > 50% was considered as significant heterogeneity.^[16]^ The funnel plot was made for observation of the potential bias and the asymmetry was tested with Egger's linear regression approach.^[17]^ All the estimate was considered significantly different when *P* < 0.05. STATA 12.0 (StataCorp., College Station, TX) was used for the meta-analysis.

## 3 Results

### 3.1 Study selection

**Figure 1:**
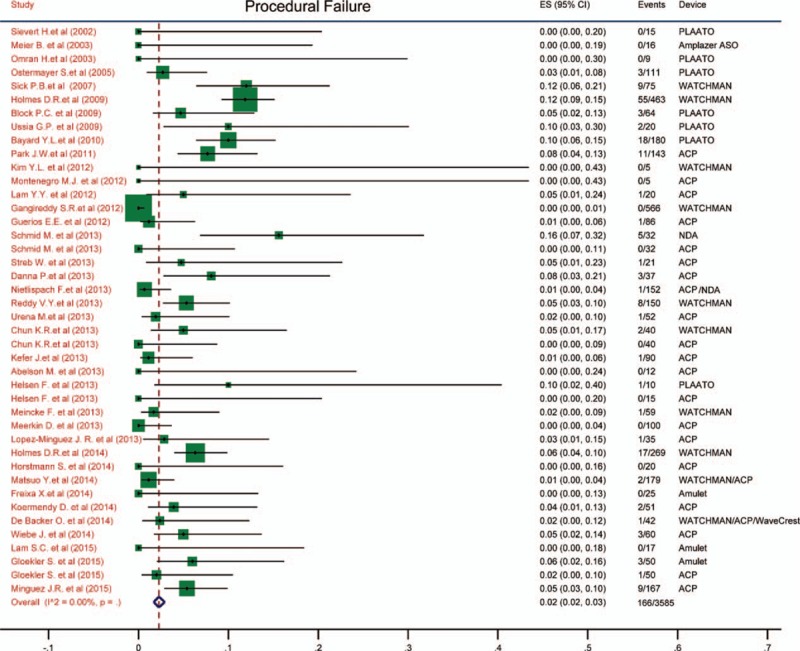
Forest plot of procedural failure rate. The marker size represented the weight of the study.

We searched 571 studies initially, 284 studies from Pubmed, 252 studies from Embase, and 35 studies from Cochrane library. In total, 138 studies were excluded due to duplicated publication or not reported in English. Among the remaining 433 studies, 335 studies were excluded for sake of comments, editorials, reviews, case reports, surgical procedure, and so on. A total of 98 studies were further screened. Also, 60 studies were excluded because of index data unaccessible or efficacy index not evaluated. Eventually, 38 studies involving 3585 patients were eligible for the predetermined criteria (Figure 1S).

### 3.2 Study quality assessment

The 38 studies included 2 RCTs (PROTECT AF and PREVAIL)^[14,15]^ and 36 observational studies, publication year from 2002 to 2015. According to Cochrane Collaberation's criteria, the 2 RCTs were both high quality and low risk of bias. Although both the RCTs design were nonblinded, the efficacy evaluation were not influenced (Table 1S). The observational studies were assessed between 5 points to 9 points using NOS criteria, which indicated they were suitable for the estimation (Table 2S).

### 3.3 Study design and characteristics

The closure systems involved in the studies were mainly Percutaneous Left Atrial Appendage Transcatheter Occlusion (PLAATO), Amplatzer Cardiac Plug device (ACP), and WATCHMAN. Other devices included nondedicated Amplatzer occluders (NDAs), Amulet, and WaveCrest. The related information and events of LAA closure were extracted and listed in Table 3S and Table 1. There were 4 studies which reported the outcomes of 2 different devices separately (Schmid et al, Helsen et al, Chun et al, and Gloekler et al).^[18–21]^ Thus, we treated them each as 2 studies when calculating the estimates. There were other 3 studies involving at least 2 closure devices (Matsuo et al, Nietlispach et al, and De Backer et al);^[22–24]^ however, the outcomes were not reported based on each device. We, therefore, took them as a single study in meta-analysis.

**Table 1:**
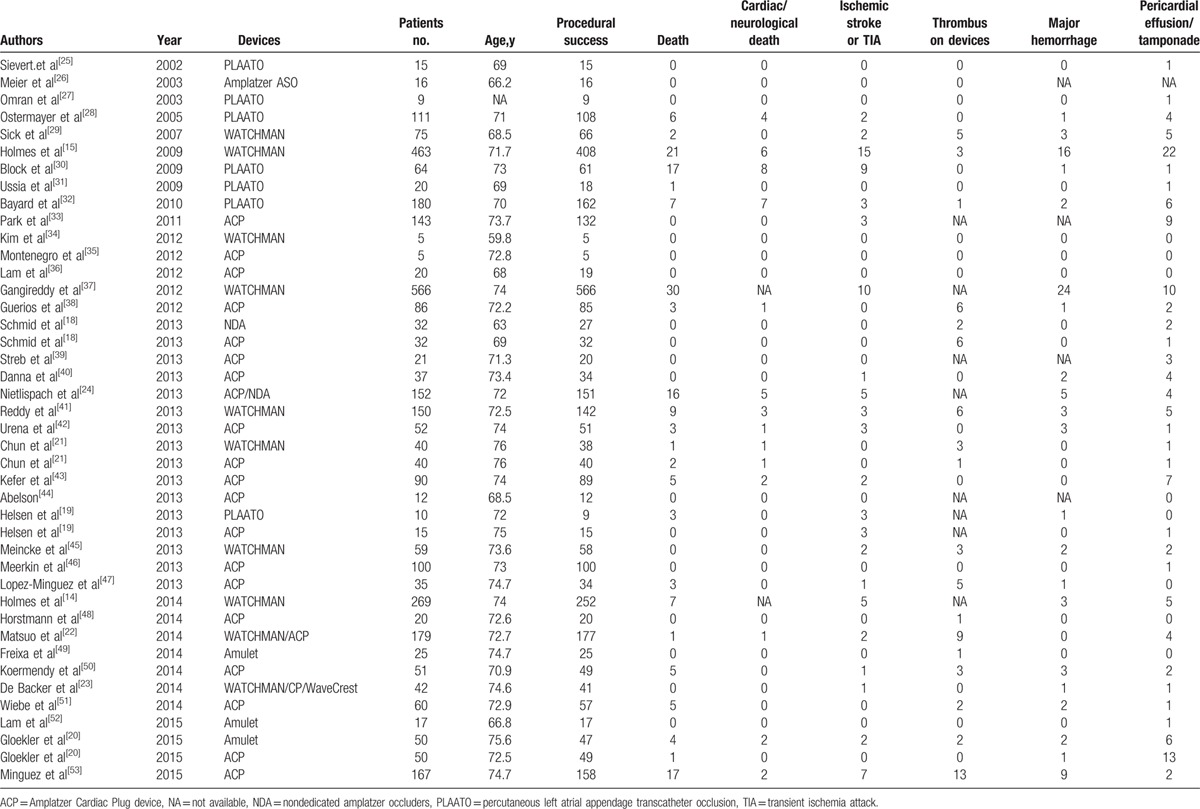
Clinical events of including studies.

### 3.4 Procedural failure

The pooled estimate of procedural failure was shown in Fig. 1. It demonstrated that the procedural failure rate of LAA closure was 0.02 (95% CI: 0.02–0.03). No heterogeneity was observed among the studies (*I*^2^* *= 0).

### 3.5 All-cause death and cardiac/neurological death

**Figure 2:**
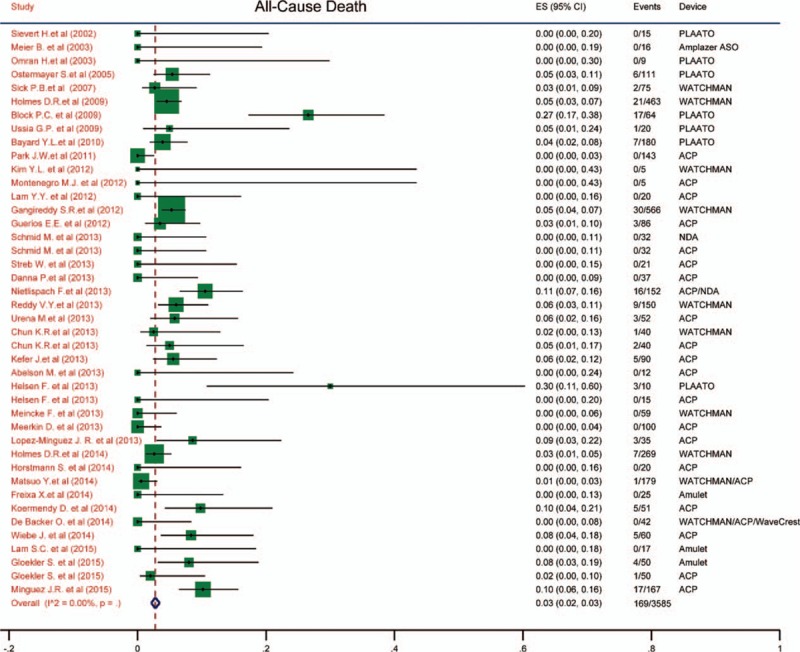
Forest plot of all-cause mortality. The marker size represented the weight of the study.

**Figure 3:**
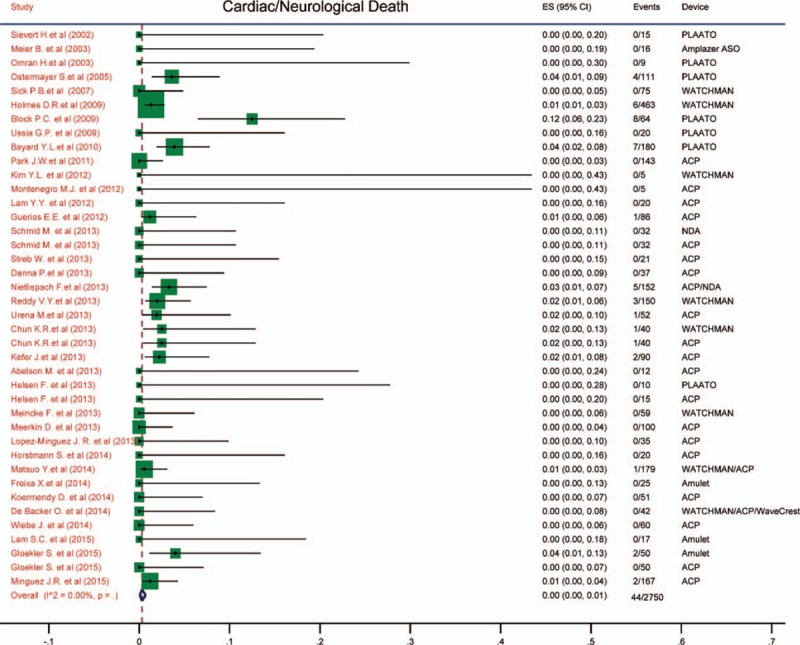
Forest plot of cardiac/neurological mortality. The marker size represented the weight of the study.

The incidence of all-cause death and cardiac/neurological death were estimated and demonstrated in Figs. 2 and 3. We found that the all-cause mortality was 0.03 (95% CI: 0.02–0.03) and cardiac/neurological mortality was 0 (95% CI: 0.00–0.01). The pooled results were quite low and there were no heterogeneity among the studies (*I*^2^ = 0).

### 3.6 Ischemic stroke/transient ischemic attack

**Figure 4:**
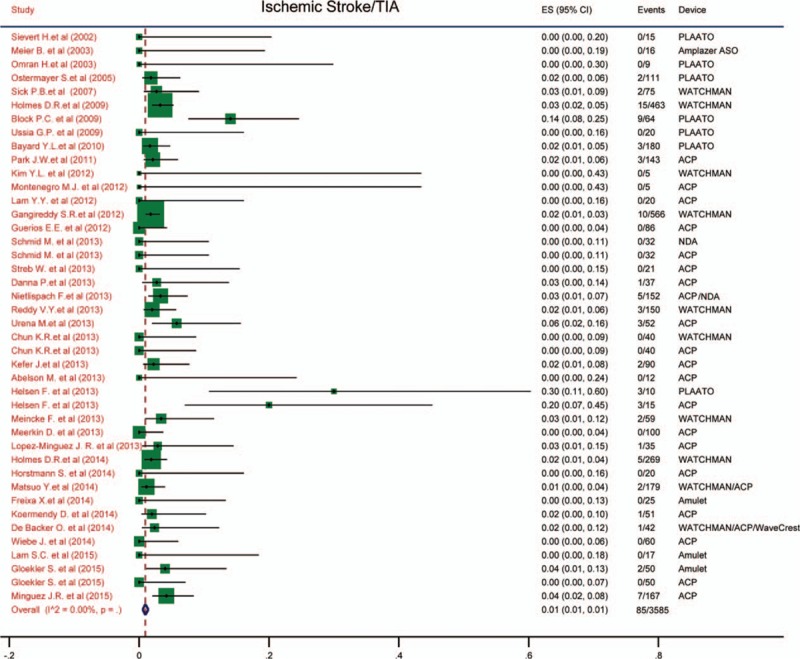
Forest plot of incidence of stroke/TIA. The marker size represented the weight of the study.

The incidence of neurological events, including ischemic stroke/transient ischemic attack (TIA) after procedure, was exhibited in Fig. 4. It revealed that the incidence of stroke/TIA was only 0.01 (95% CI: 0.01–0.01). There was no heterogeneity among the pooled studies (*I*^2^ = 0).

### 3.7 Thrombus on devices

**Figure 5:**
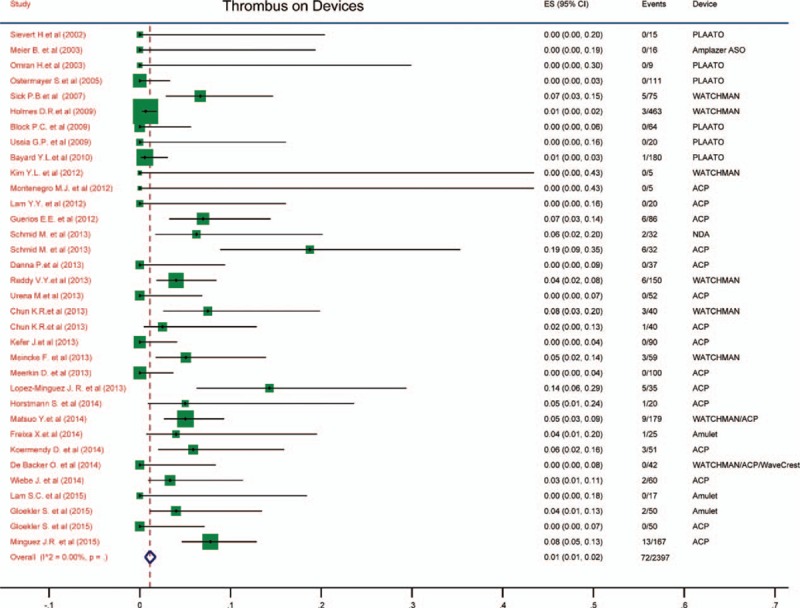
Forest plot of incidence of thrombus on devices. The marker size represented the weight of the study.

Thrombus on the occlusion devices was also a significant event after LAA transcatheter closure. The incidence of thrombus on devices was 0.01 (95% CI: 0.01–0.02), which was shown in Fig. 5. No heterogeneity was observed as well (*I*^2^ = 0).

### 3.8 Major hemorrhagic complications

The estimated incidence of major hemorrhagic complications was shown in Figure 2S. The major hemorrhagic complications rate were estimated 0.01 (95% CI: 0.00–0.01). There were also no heterogeneity (*I*^2^ = 0).

### 3.9 Pericardial effusion/cardiac tamponade

Pericardial effusion is common in the periprocedural period, which would probably risk patients’ lives when it leads to tamponade. In Figure 3S, it was demonstrated that the incidence of this complication was 0.02 (95% CI: 0.02–0.03) and there was no heterogeneity alike (*I*^2^ = 0).

### 3.10 Subgroup analysis

PLAATO, WATCHMAN, and ACP were the most popular occlusion devices. The subgroup analysis was performed to compare the differences in the events among the 3 devices (Table 2). Consequently, the all-cause mortality and cardiac/neurological mortality in the PLAATO group were the highest among the 3 devices (*P* = 0.01 and *P* < 0.01, respectively). Besides, the incidence of thrombus on devices in the ACP group was the highest and that in the PLAATO group was the lowest (*P* < 0.01). No significant differences were observed in other events among the 3 groups.

**Table 2:**
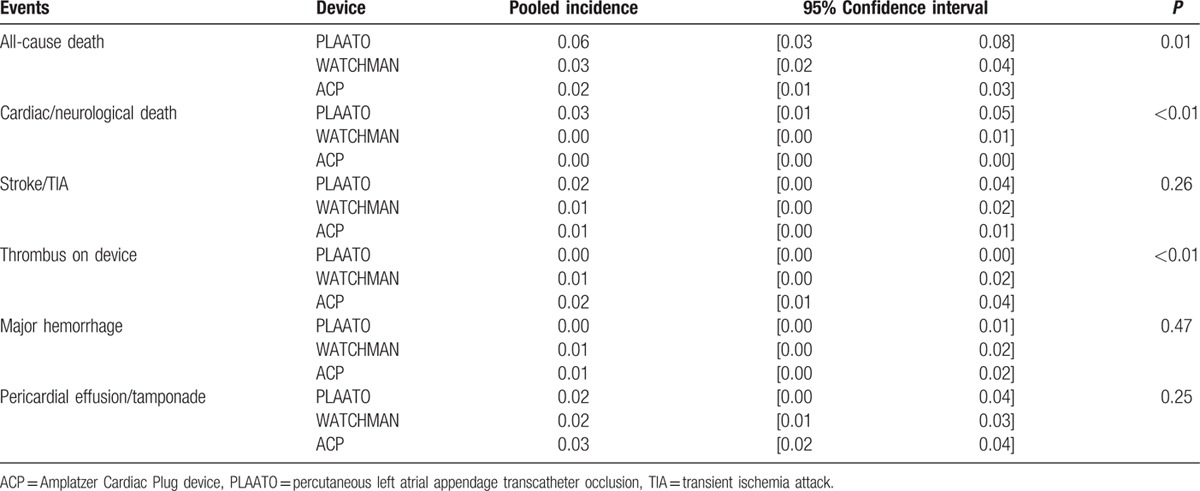
Efficacy and safety comparison in different occlusion devices.

We also considered the possible impact of follow-up period on the events and, therefore, assessed the differences between the follow-up period <12 months and >12 months (Table 3). There were significant differences in all-cause death, stroke/TIA, major hemorrhage, and pericardial effusion/tamponade events (*P* < 0.05) between the 2 subgroups. No difference in thrombus on devices was observed (*P* = 0.53).

**Table 3:**
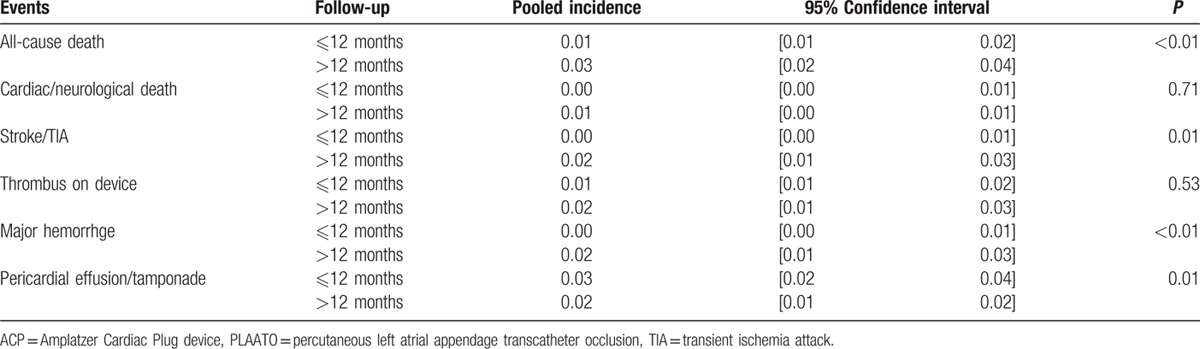
Efficacy and safety comparison at different follow-up periods.

### 3.11 Funnel plot

The funnel plot with all-cause death versus its standard error of including study was shown in Figure 4S. The asymmetry test with Egger's liner regression revealed intercept was −0.07 with 95% CI: −0.32–0.19 (*P* = 0.61), which meant the funnel plot was statistically symmetrical.

## 4 Discussion

It is necessary to balance the ischemic and hemorrhagic risk, such as individual characteristic, drug tolerability, treatment compliance, when the doctors make a strategy of stroke prevention for AF patients. Several NOACs have been proved noninferior effect to VKA but with better safety, especially in terms of intracranial hemorrhage. Thus, NOACs are more suitable for the patients with labile international normalized ratio (INR) or who cannot or will not monitor INR. However, there are ∼30% to 50% patients with contraindication to long-term use of anticoagulant.^[54]^ The development of transcatheter LAA occlusion provides a new approach to the patients who are not suitable for lifelong antithrombotic therapy. Although the application of this procedure is reasonable and feasible, most data about the efficacy and safety of LAA occlusion are provided by observational studies apart from the 2 existing RCTs. At present, the recommendation of LAA occlusion is IIb in 2012 guideline.^[55]^

After combination of the data from the past decade, we found the procedural failure rate was only 2%, although this procedure was complex. The progress of LAA occlusion benefits from the advanced technology of radiology and ultrasound as well as the improvement of devices. The all-cause mortality and cardiac/neurological mortality were also low, which was an inspiring consequence for both doctors and patients. In stroke risk assessment, patients with CHA_2_DS_2_-VASc score >1 point are usually recommended to antithrombotic therapy. The stroke rate was from 1.3% at 1 point to 15.2% at 9 points.^[56]^ The estimated stroke/TIA rate in the current study was 1%, which was similar to the stroke risk at CHA_2_DS_2_-VASc score of 1 point. Meanwhile, the estimated incidence of thrombus on devices was 1%, which was a potential risk of neurological events after procedure. Of note, most patients included in the meta-analysis had the indication of antithrombotic therapy but also had contraindications or intolerability to long-term use of anticoagulant. The postprocedural medical regimens were mainly aspirin plus clopidogrel and/or plus short-term use of warfarin. It was evident that LAA closure would not cause increased stroke/TIA rate due to possible thrombus on the devices despite of antiplatelet therapy after LAA occlusion. In regard to major hemorrhagic complication, it was revealed also quite low in our study. In contrast, the major bleeding rate of anticoagulant was from 2% to >3.3% in clinical studies.^[57,58]^ As such, LAA occlusion is seemed to be a procedure of low hemorrhagic risk. Pericardial effusion or tamponade, as an important periprocedural complication, sometimes is fatal. This adverse event was considered due to inappropriate trans-septal puncture, device oversize, violent manipulation of catheter, and so on. However, the incidence of this complication was only 2%, namely, the risk of pericardial effusion/tamponade could be controlled with prudent manipulation in the procedure.

In subgroup analysis, we found that the all-cause mortality and cardiac/neurological mortality were highest in the PLAATO group, whereas the incidence of thrombus on devices was the lowest in this group. PLAATO was the first system developed for LAA closure. Compared with following system, such as WATCHMAN, ACP, PLAATO was less flexible and therefore replaced by the latter.^[59]^ Most of the studies related with PLAATO were performed before 2011, whereas WATCHMAN and ACP were the widely used in recent years. The higher mortality of PLAATO was probably attributed to the operators’ experiences and learning curve.

It was reported that the incidence of residual flow in LAA after implantation of WATCHMAN was up to >30%,^[60]^ which was likely to increase the neurological events after the procedure theoretically. By contrast, the ACP system had an additional disk, which made higher possibility to complete occlusion. However, the incidence of stroke/TIA was quite low and similar among the 3 devices. Despite of the significant difference on the incidence of thrombus on devices, the rates were numerically small. Thus, the difference among the subgroups was seemed to be of little clinical value.

The EWOLUTION registry study has been published recently. In this study, the CHA_2_DS_2_-VASc score of the patients was average 4.5 points and HAS-BLED score was average 2.3 points. Of note, 40% patients had HAS-BLED score ≥3 points. The results have confirmed the high success rate and low peri-procedural complication, particularly quite low incidence of stroke and hemorrhagic events of WATCHMAN device in the patients with high stroke risk as well as high hemorrhagic risk.^[61]^ The favorable consequences largely attributed to the improvement of the procedural technique, which implied that the learning curve was more important that the structural design of the devices.

We also further analyzed the impact of follow-up period on the efficacy and safety outcomes. We found the death rate of all-cause in the group of follow-up period >12 months was significantly higher, whereas the cardiac/neurological death was not. Many of the deaths in late phase of follow-up were not cardiac/neurological or not procedural-related, such as trauma, cancer, aortic dissection.^[21,29,31]^ As to major hemorrhage, the definitions were not consistent among the studies. But the major hemorrhage was mainly due to periprocedural complications and postprocedural antithrombotic regimens. It was believed that the higher incidence of major hemorrhage in longer follow-up period group was probably due to long-term use of antithrombotic regimens. More importantly, whether there was significant difference between the shorter and longer follow-up period subgroups or not, the incidences of the events were all extremely low. So to speak, the safety events were not likely to increase apparently as the follow-up period prolonged.

## 5 Limitations

There were several limitations in the current study. First, the definitions of the efficacy and safety endpoints were more or less different in the including studies, which would bias the estimated results. Second, cardiac/neurological death was considered as a critical index for efficacy and safety evaluation. Two studies with large sample size did not report the incidence of cardiac/neurological death,^[14,37]^ which led to a great loss of information. Third, it should be noticed that in subgroup analysis, the number of PLAATO studies and involved patients were the least, whereas the number of WATCHMAN and ACP studies and their involved patients were much more. The imbalance of the sample size would possibly produce deviations to the pooled estimate, although the funnel plot and Egger's test supported no publication bias. The last but not the least, some studies reported the outcomes of different occlusion devices as a whole, which made us impossible to extract the data of the single device. Consequently, these data could not be utilized in subgroup analysis.

## 6 Conclusions

The current meta-analysis identified the excellence of efficacy and safety outcomes and extremely low rate of procedural failure in transcatheter LAA occlusion. Subgroup analysis found that the differences among the 3 occlusion devices and the differences between shorter and longer follow-up period were so small that it did not make sense in terms of clinical practice.

Considered the efficacy and safety of LAA occlusion, this technique has been recommended for the AF patients who were not suitable for long-term use of anticoagulant both in European and American guidelines.^[4,55]^ However, the class of recommendation was still IIb in European guideline, whereas there was no clear class of recommendation in American guideline. It was so prudent mainly because of less evidence from RCTs. It is believed that as the implantation technique improves and more relevant studies emerge, the scenery will probably get better.

## Supplementary Material

Supplemental Digital Content
