# Supplementary material for: A meta-analysis for efficacy and safety evaluation of transcatheter left atrial appendage occlusion in patients with nonvalvular atrial fibrillation
Source: Medicine (Baltimore). 2016 Aug 7;95(31):e4382. doi: 10.1097/MD.0000000000004382 (PMC4979802; doi:10.1097/MD.0000000000004382)
Supplement: Supplemental Digital Content [file medi-95-e4382-s001.docx]

Figure 1S. Flow diagram of the studies selection progress

Figure 2S. Forest plot of incidence of major hemorrhage. The marker size represented the weight of the study.





Figure 3S. Forest plot of incidence of pericardial effusion/tamponade. The marker size represented the weight of the study.


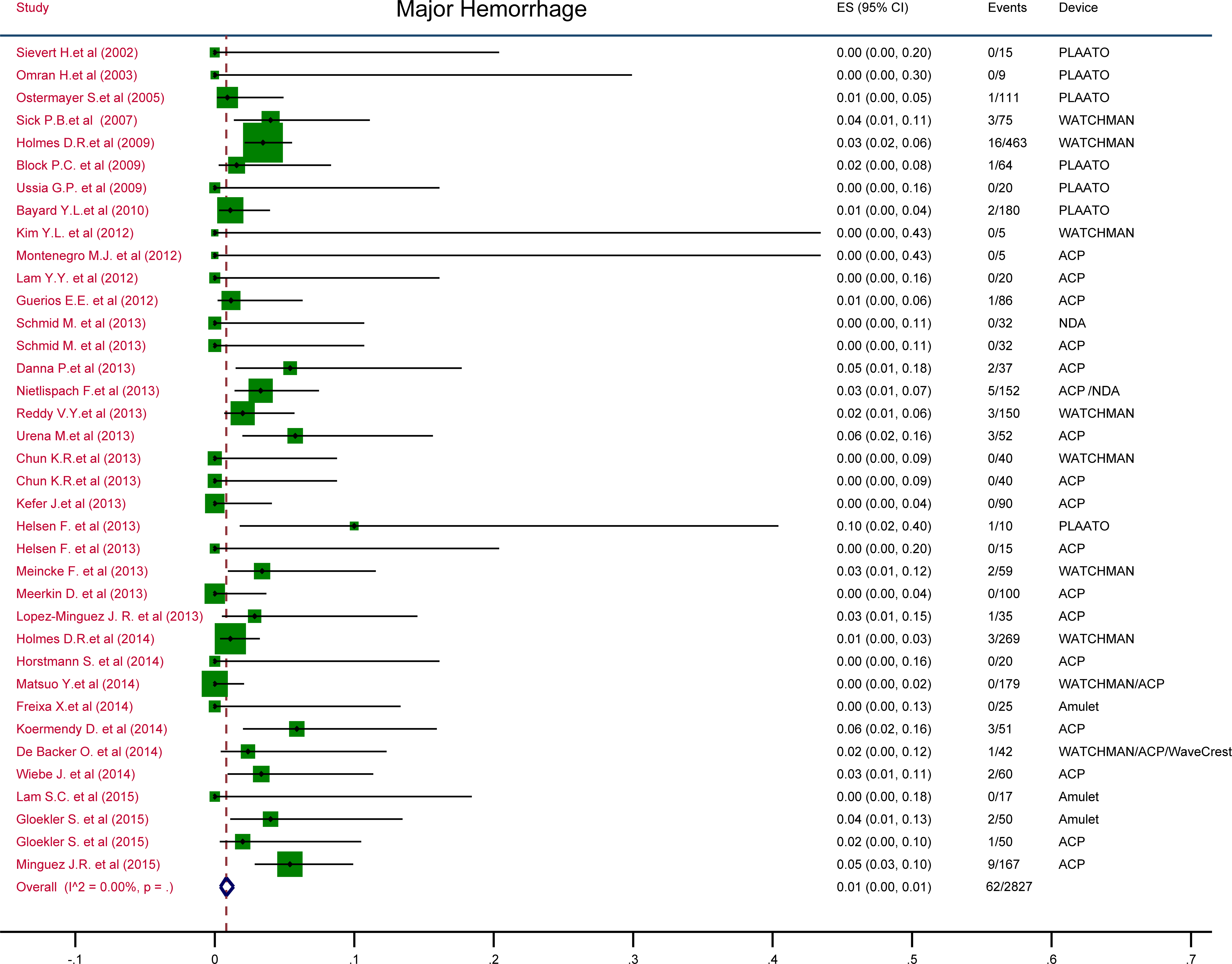


Figure 4S. Funnel plot analysis for effect size of all-cause mortality. The plot was made with the all-cause mortality versus the precision of the study.


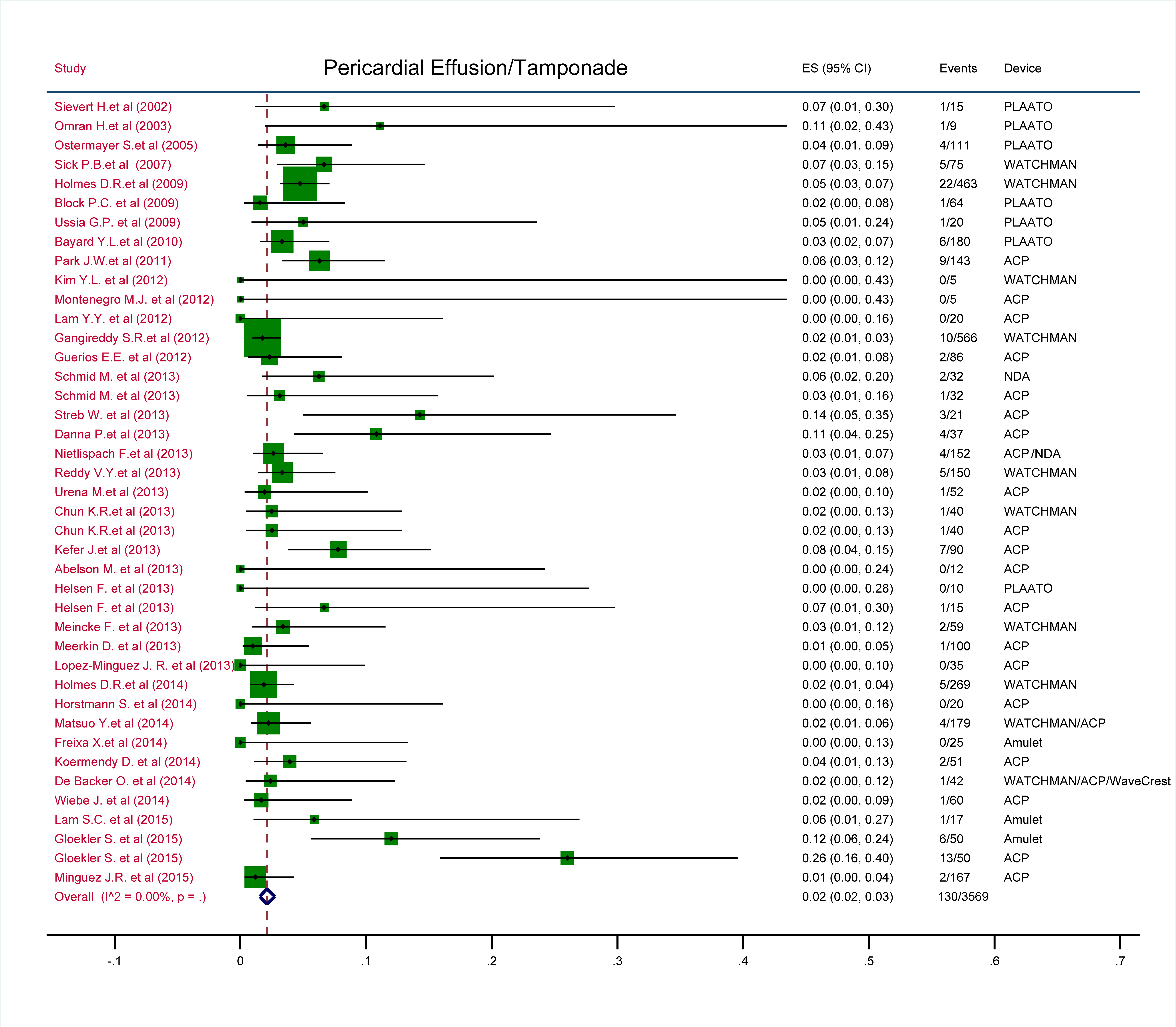

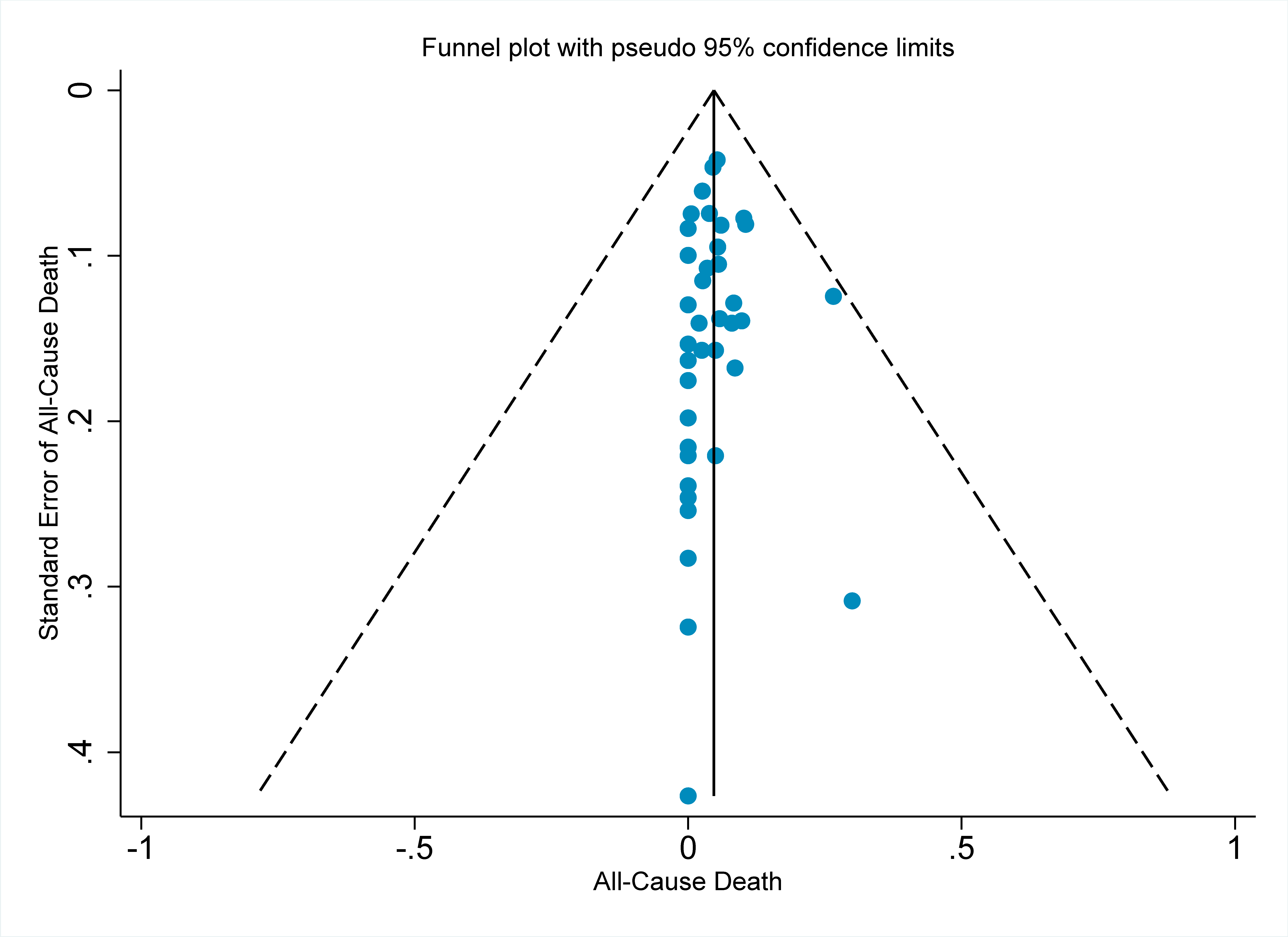


| **Table 1S. Quality assessment of RCTs** | | | | | | | | | |
| --- | --- | --- | --- | --- | --- | --- | --- | --- | --- |
| **Study** | **Authors** | **Year** | **Devices** | **Sequence  generation** | **Allocation  concealment** | **Blinding** | **Incomplete outcome data** | **Selective outcome reporting** | **Other source of bias** |
| PROTECT AF | Holmes D.R.et al | 2009 | WATCHMAN | Yes | Yes | No | Yes | No | No |
| PREVAIL | Holmes D.R.et al | 2014 | WATCHMAN | Yes | Yes | No | Yes | No | No |

RCT: Randomized Controlled Trial

| **Table 2S. Quality assessment of observational studies** | | | |
| --- | --- | --- | --- |
| **Authors** | **Year** | **Devices** | **NOS score** |
| Sievert H.et al | 2002 | PLAATO | 5 |
| Meier B. et al | 2003 | Amplazer ASO | 5 |
| Omran H.et al | 2003 | PLAATO | 6 |
| Ostermayer S.et al | 2005 | PLAATO | 6 |
| Sick P.B.et al | 2007 | WATCHMAN | 6 |
| Block P.C. et al | 2009 | PLAATO | 6 |
| Ussia G.P. et al | 2009 | PLAATO | 6 |
| Bayard Y.L.et al | 2010 | PLAATO | 6 |
| Park J.W.et al | 2011 | ACP | 5 |
| Kim Y.L. et al | 2012 | WATCHMAN | 5 |
| Montenegro M. J. et al | 2012 | ACP | 6 |
| Lam Y.Y. et al | 2012 | ACP | 6 |
| Gangireddy S.R.et al | 2012 | WATCHMAN | 6 |
| Guerios E.E. et al | 2012 | ACP | 6 |
| Schmid M. et al | 2013 | NDA/ACP | 7 |
| Streb W. et al | 2013 | ACP | 5 |
| Danna P.et al | 2013 | ACP | 6 |
| Nietlispach F.et al | 2013 | ACP/NDA | 7 |
| Reddy V.Y.et al | 2013 | WATCHMAN | 6 |
| Urena M.et al | 2013 | ACP | 6 |
| Chun K.R.et al | 2013 | WATCHMAN/ACP | 9 |
| Kefer J.et al | 2013 | ACP | 6 |
| Abelson M. et al | 2013 | ACP | 6 |
| Helsen F. et al | 2013 | PLAATO/ACP | 9 |
| Meincke F. et al | 2013 | WATCHMAN | 6 |
| Meerkin D. et al | 2013 | ACP | 5 |
| Lopez-Minguez J. R. et al | 2013 | ACP | 6 |
| Horstmann S. et al | 2014 | ACP | 6 |
| Matsuo Y.et al | 2014 | WATCHMAN/ACP | 6 |
| Freixa X.et al | 2014 | Amulet | 5 |
| Koermendy D. et al | 2014 | ACP | 5 |
| De Backer O. et al | 2014 | WATCHMAN/ ACP/WaveCrest | 6 |
| Wiebe J. et al | 2014 | ACP | 6 |
| Lam S.C. et al | 2015 | Amulet | 5 |
| Gloekler S. et al | 2015 | Amulet/ACP | 9 |
| Minguez J.R. et al | 2015 | ACP | 6 |

PLAATO: Percutaneous Left Atrial Appendage Transcatheter Occlusion; ACP: Amplatzer Cardiac Plug device; NDA: Non-Dedicated Amplatzer occluders; NOS: Newcastle-Ottawa Scale

| **Table 3S. Characteristics of including studies** | | | | | | | | | | | | | |
| --- | --- | --- | --- | --- | --- | --- | --- | --- | --- | --- | --- | --- | --- |
| **Authors** | **Year** | **Study type** | **Follow-up period** | **Devices** | **Device  size, mm** | | | **Procedural  time,min** | | **Indication** | | **Postprocedural medical regimens** | |
| Sievert H.et al^25^ | 2002 | observational | 1 month | PLAATO | | 20.1 | 92.7 | | contraindication to long-term  anticoagulant | | aspirin 300mg/d for long-term+clopidogrel 75mg/d for 6 months | |  |
| Meier B. et al^26^ | 2003 | observational | 4 months | Amplatzer ASO | | 15.7 | NA | | not suitable or refusal to anticoagulant | | aspirin+clopidogrel for 3-6 months, warfarin if necessary | |  |
| Omran H.et al^27^ | 2003 | observational | 5 months | PLAATO | | NA | 82 | | contraindication to  anticoagulant | | aspirin+clopidogrel for 3 months | |  |
| Ostermayer S.et al^28^ | 2005 | observational | 9.8 month | PLAATO | | 29 | 68 | | contraindication to  anticoagulant | | aspirin 300-325mg/d for long-term+clopidogrel 75mg/d for  4-6 weeks | |  |
| Sick P.B.et al^29^ | 2007 | observational | 24 months | WATCHMAN | | 24 | NA | | eligible for anticoagulant | | aspirin 81-100mg+warfarin for 45 days | |  |
| Holmes D.R.et al^15^ | 2009 | RCT | 18 months | WATCHMAN | | NA | NA | | eligible for anticoagulant | | warfarin for 45 days,followed by aspirin 81-325mg/d+clopidogrel  75mg/d until 6 months,then aspirin for long-term | |  |
| Block P.C. et al^30^ | 2009 | observational | 5 years | PLAATO | | NA | NA | | contraindication to long-term  anticoagulant | | aspirin 325mg/d for long-term+clopidogrel 75mg for 4-6 weeks | |  |
| Ussia G.P. et al^31^ | 2009 | observational | 40 months | PLAATO | | 28 | NA | | contraindication or refusal  to anticoagulant | | aspirin 160mg/d+ticlopidine 250mg bid for 6 months | |  |
| Bayard Y.L.et al^32^ | 2010 | observational | 9.6 months | PLAATO | | 29 | 65 | | contraindication to long-term  anticoagulant | | aspirin 300-325mg/d for long-term+clopidogrel 75mg/d for  6 months | |  |
| Park J.W.et al^33^ | 2011 | observational | 24 hours | ACP | | 22.5 | NA | | NA | | NA | |  |
| Kim Y.L. et al^34^ | 2012 | observational | NA | WATCHMAN | | 28.8 | 72.8 | | not tolerate to long-term  anticoagulant | | aspirin 100mg/d+warfarin for 8 weeks, aspirin 100mg/d +clopidogrel 75mg/d thereafter | |  |
| Montenegro M. J. et al^35^ | 2012 | observational | 9 month | ACP | | 25.2 | NA | | not suitable for  anticoagulant | | antiplatelet therapy not in details | |  |
| Lam Y.Y. et al^36^ | 2012 | observational | 12.7 months | ACP | | 23.6 | NA | | contraindication to  anticoagulant | | aspirin 80-160mg/d for long-term+clopidogrel 75mg/d for 1 month | |  |
| Gangireddy S.R.et al^37^ | 2012 | observational | 16 months | WATCHMAN | | NA | NA | | eligible for anticoagulant | | warfarin for 45 days,followed by aspirin 81-325mg/d+clopidogrel  75mg/d until 6 months, then aspirin for long-term | |  |
| Guerios E.E. et al^38^ | 2012 | observational | 3.7 months | ACP | | 23.1 | NA | | contraindication or aversion to long-term anticoagulant | | aspirin 100mg/d for 3-4 months+clopidogrel 75mg/d for 1 months | |  |
| Schmid M. et al^18^ | 2013 | observational | 7.2 months | NDA/ACP | | NA | NA | | physician or patients refusal  of anticoagulant | | antiplatelet or anticoagulant therapy, not in details | |  |
| Streb W. et al^39^ | 2013 | observational | NA | ACP | | 23.9 | 103.1 | | contraindication to anticoagulant or recurrence of stroke despite of OAC therapy | | aspirin for at least 6 months+clopidogrel 75mg/d for 1-3 months | |  |
| Danna P.et al^40^ | 2013 | observational | 12 months | ACP | | 23.7 | 92.9 | | contraindication to long-term  anticoagulant | | aspirin for at least 6 months+clopidogrel for at least 1 month | |  |
| Nietlispach F.et al^24^ | 2013 | observational | 32 months | ACP/NDA | | NA | NA | | eligible for anticoagulant | | aspirin 100mg/d+clopidogrel 75mg/d for 1-6 month, OAC for  3 months if underwent LA ablation | |  |
| Reddy V.Y.et al^41^ | 2013 | observational | 14.4 months | WATCHMAN | | 26 | 51.5 | | contraindication to anticoagulant | | aspirin 81-325mg/d+warfarin for 45 days,followed by aspirin  81-325mg for long-term+clopidogrel 75mg/d for 6 months | |  |
| Urena M.et al^42^ | 2013 | observational | 20 months | ACP | | 23.5 | NA | | contraindication to  anticoagulant | | aspirin 80-325mg/d+clopidogrel 75mg/d,or aspirin or clopidogrel  for 1-6 months according to operator's discretion, single antiplatelet therapy for long-term | |  |
| Chun K.R.et al^21^ | 2013 | observational | 1 year | WATCHMAN | | 24.7 | 48 | | contraindication or refusal  to anticoagulant | | aspirin 100mg/d+clopidogrel 75mg/d or OAC for 6 weeks,aspirin  100mg/d for long-term | |  |
| Chun K.R.et al^21^ | 2013 | observational | 1 year | ACP | | 23.8 | 47 | | contraindication or refusal  to anticoagulant | | aspirin 100mg/d+clopidogrel 75mg/d or OAC for 6 weeks,aspirin  100mg/d for long-term | |  |
| Kefer J.et al^43^ | 2013 | observational | 1 year | ACP | | 24.5 | NA | | contraindication to  anticoagulant | | dual or single antiplatelet therapy or OAC according to  physician's discretion | |  |
| Abelson M. et al^44^ | 2013 | observational | 12.8 months | ACP | | 25.8 | NA | | contraindication to  anticoagulant | | aspirin 82-150mg for long-term+clopidogrel 75mg/d for 1 month | |  |
| Helsen F. et al^19^ | 2013 | observational | 4.8 years | PLAATO | | NA | NA | | relative contraindication  to anticoagulant | | aspirin 160mg bid+clopidogrel 75mg/d for 6 months, aspirin 160mg for long-term | |  |
| Helsen F. et al^19^ | 2013 | observational | 1.1 years | ACP | | NA | NA | | relative contraindication  to anticoagulant | | aspirin 160mg qd for 6 months+ clopidogrel 75mg/d for 3 months, aspirin 80mg/d for long-term | |  |
| Meincke F. et al^45^ | 2013 | observational | 6 months | WATCHMAN | | 26.8 | NA | | contraindication to  anticoagulant | | aspirin 100mg/d for long-term+clopidogrel 75mg/d for 6 months | |  |
| Meerkin D. et al^46^ | 2013 | observational | NA | ACP | | 24.3 | NA | | contraindication to long-term anticoagulant | | aspirin 300mg loading dose,100mg/d for long-term+clopidogrel  300mg loading dose,75mg/d for 1 months. | |  |
| Lopez-Minguez J. R.et al^47^ | 2013 | observational | 21.1 months | ACP | | 22.1 | 92.5 | | contraindication to anticoagulant | | aspirin 300mg for 1 day,100mg/d for 6 months+clopidogrel  75mg/d for 3 months | |  |
| Holmes D.R.et al^14^ | 2014 | RCT | 11.8 months | WATCHMAN | | NA | NA | | eligible for anticoagulant | | aspirin 81mg/d+warfarin for 45 days,then aspirin 81-325mg/d  for long-term and clopidogrel 75mg/d until 6 months | |  |
| Horstmann S. et al^48^ | 2014 | observational | 13.6 months | ACP | | NA | NA | | with ICH history | | aspirin 100mg/d for long-term+clopidogrel 75mg/d for 3 months | |  |
| Matsuo Y.et al^22^ | 2014 | observational | 6 months | WATCHMAN /ACP | | NA | 33.9 | | contraindication to long-term  anticoagulant | | one of the following regimens:1.warfarin for at least 45 days; 2.dabigtran 150mg bid for 45 days,followed by aspirin 100mg/d  for long-term+clopidogrel 75mg/d for 4.5 months;3.aspirin 100mg/d for long-term +clopidogrel 75mg/d for 6 months; 4.replacement of dabigtran with enoxaparin 0.01mg/kg bid sc.in regimen 2 | |  |
| Freixa X.et al^49^ | 2014 | observational | 3 months | Amulet | | 25 | NA | | contraindication to  anticoagulant | | aspirin 80mg/d for long-term+clopidogrel 75mg/d for at least 3 months | |  |
| Koermendy D. et al^50^ | 2014 | observational | 4.6 months | ACP | | 25 | 80.6 | | not suitable for or patients  refusal of anticoagulant | | aspirin 100mg/d+clopidogrel 75mg/d for 1-6 months | |  |
| De Backer O. et al^23^ | 2014 | observational | 12.6 months | WATCHMAN/ ACP/WaveCrest | | NA | NA | | contraindication to  anticoagulant | | NA | |  |
| Wiebe J. et al^51^ | 2014 | observational | 1.8 years | ACP | | NA | 63.7 | | contraindication to  anticoagulant | | aspirin 100mg/d for long-term+clopidogrel 75mg/d for 3 months | |  |
| Lam S.C. et al^52^ | 2015 | observational | 3 months | Amulet | | 25.5 | NA | | not suitable for  anticoagulant | | aspirin for at least 6 months+clopidogrel 75mg/d for 3 month | |  |
| Gloekler S. et al^20^ | 2015 | observational | 3.5 months | Amulet | | 28.3 | NA | | eligible for anticoagulant | | aspirin 100mg/d+clopidogrel 75mg/d for at least 1 month | |  |
| Gloekler S. et al^20^ | 2015 | observational | 4.2 months | ACP | | 27.8 | NA | | eligible for anticoagulant | | aspirin 100mg/d+clopidogrel 75mg/d for at least 2 month | |  |
| Minguez J.R. et al^53^ | 2015 | observational | 22 months | ACP | | 24 | NA | | contraindication or  inappropriate to anticoagulant | | aspirin 300mg loading dose,100mg/d for long-term+clopidogrel  600mg loading dose,75mg/d for 6-12 months. | |  |

PLAATO: Percutaneous Left Atrial Appendage Transcatheter Occlusion; ACP: Amplatzer Cardiac Plug device; NDA: Non-Dedicated Amplatzer occluders; RCT: Randomized Controlled Trial; NA: Not Available
